# Incidence and risk factors of new persistent opioid use after surgery and trauma: A systematic review

**DOI:** 10.1186/s12893-024-02494-0

**Published:** 2024-07-16

**Authors:** Jiayi Gong, Peter Jones, Amy Hai Yan Chan

**Affiliations:** 1 School of Pharmacy, Faculty of Medical and Health Sciences, The University of Auckland, Auckland, New Zealand; 2 Department of Surgery, Faculty of Medical and Health Sciences, The University of Auckland, Auckland, New Zealand

**Keywords:** Surgery, Trauma, Opioids, Persistent opioid use

## Abstract

**Background:**

Persistent opioid use (POU) can occur with opioid use after surgery or trauma. Current systematic reviews include patients with previous exposure to opioids, meaning their findings may not be relevant to patients who are opioid naïve (i.e. Most recent exposure was from surgery or trauma). The aim of this review was to synthesise narratively the evidence relating to the incidence of, and risk factors for POU in opioid-naïve surgical or trauma patients.

**Method:**

Structured searches of Embase, Medline, CINAHL, Web of Science, and Scopus were conducted, with final search performed on the 17th of July 2023. Searches were limited to human participants to identify studies that assessed POU following hospital admission due to surgery or trauma. Search terms relating to ‘opioid’, ‘analgesics’, ‘surgery’, ‘injury’, ‘trauma’ and ‘opioid-related disorder’ were combined. The Newcastle–Ottawa Scale for cohort studies was used to assess the risk of bias for studies.

**Results:**

In total, 22 studies (20 surgical and two trauma) were included in the analysis. Of these, 20 studies were conducted in the United States (US). The incidence of POU for surgical patients 18 and over ranged between 3.9% to 14.0%, and for those under 18, the incidence was 2.0%. In trauma studies, the incidence was 8.1% to 10.5% among patients 18 and over. Significant risk factors identified across surgical and trauma studies in opioid-naïve patients were: higher comorbidity burden, having pre-existing mental health or chronic pain disorders, increased length of hospital stay during the surgery/trauma event, or increased doses of opioid exposure after the surgical or trauma event. Significant heterogeneity of study design precluded meta-analysis.

**Conclusion:**

The quality of the studies was generally of good quality; however, most studies were of US origin and used medico-administrative data. Several risk factors for POU were consistently and independently associated with increased odds of POU, primarily for surgical patients. Awareness of these risk factors may help prescribers recognise the risk of POU after surgery or trauma, when considering continuing opioids after hospitalisation. The review found gaps in the literature on trauma patients, which represents an opportunity for future research.

**Trial registration:**

PROSPERO registration: CRD42023397186.

**Supplementary Information:**

The online version contains supplementary material available at 10.1186/s12893-024-02494-0.

## Background

Persistent opioid use (POU) in patients who have been admitted to hospital for surgery or trauma is a growing area of interest, due to the common use of opioids as a mainstay treatment of acute pain [1, 2]. POU is defined in most studies as continued opioid use beyond 90 days after surgery or trauma with the definition adapted from diagnostic criteria for chronic pain [3, 4]. Much of the literature on POU has been in the post-surgical context, with few studies in trauma, and even fewer focusing on risk of POU development in opioid-naïve patients [5, 6]. Previous systematic reviews have mostly focused on patients regardless of any recent exposures to opioids prior to surgery [5–7]. Patients with a recent history of opioid use before hospital admission may indicate chronic opioid usage, and have important differences in terms of risk factors for POU characteristics and opioid needs compared to opioid-naïve patients [8–10]. Patients with prior opioid use and subsequent continuation of opioids after hospitalisation may also represent opioid use for therapeutic indications related to non-surgical and non-trauma indications [3]. This represents an important gap in the literature, and a review focusing on opioid-naïve patients after surgery or trauma would be informative.

POU may be a useful marker in detecting harm such as opioid misuse and mortality after surgery or trauma [3]. A recent study from the United States (US) by *Santosa *et al*.* reported persistent opioid users after surgery have a higher risk of mortality (Hazard Ratio (HR) 3.44; 95% confidence interval (CI) 2.99–3.96) and opioid-related readmissions and visits to the emergency department (adjusted odds ratio (aOR) 1.68; 95% CI 1.55–1.82) after adjusting for important clinical and demographic covariates [11]. Thus, identifying risk factors related to POU may be key for informing policymakers and stakeholders in minimising and preventing harm related to POU.

The aim of this systematic review is to synthesise the current literature on POU in opioid-naive patients after surgery or trauma and risk factors for POU.

## Method

### Search strategy

A systematic search was first carried out on the 18th of January 2023 and a final search was carried out on the 17th of July 2023. A structured search of the following databases was undertaken using the OVID platform for Embase (1980 to current) and Medline (1946 to current), Cumulative Index to Nursing and Allied Health Literature (CINAL Plus) hosted on the EBSCO website from 1937 to the present, Web of Science provided by Clarivate, and Scopus. Previously published reviews on the topic were used to inform the search strategy and search terms. Reference lists of previous studies were used to identify relevant studies via the snowballing (pearl-growing) method [12]. Keywords relating to opioids, surgery, trauma, analgesia, persistent opioid and opioid dependence were used to construct the search strategy. Reporting of this review was according to 2020 PRISMA statement [13]. The specific search terms and strategies for the respective databases are in the supplementary material- Part 1.

Searches were limited to human participants. No limits were placed on the publication date. Conference proceedings and grey literature were excluded.

### Study selection

The following inclusion criteria were used for selection of studies in this systematic review: 1) the study included patients that were admitted to the hospital with trauma or had undergone surgery; 2) all patients included in the primary analysis were opioid-naïve e.g. no recent exposure to opioids, as defined by the study; 3) POU must be measured as an outcome – defined as *any* initial opioid exposure following the surgery or trauma event around 30 days of the event (either on discharge or before the event), then subsequent re-exposure to *any* opioids from 90 days after the event; and 4) the duration of assessment of POU does not exceed 365 days after the event. There must be only two time periods associated with the definition of POU, one for initiation related to the event (30 days prior to and, up to 30 days after the event) and subsequent re-exposure (90 days after the event, up to 365 days). The rationale for these two time periods was that without the initial opioid exposure immediately before or after the event, subsequent opioid prescription may be unrelated to either surgery or trauma and does not represent POU. In addition, any additional criteria on opioid use will result in exclusion as per below.

We excluded the following types of studies:

- Studies with a POU definition that included any criteria specifying a threshold for a number of prescriptions dispensed or filled by a pharmacy during any of the two main time points e.g. need to fill more than one prescription at either time points.
- Studies with a POU definition that included criteria specifying a threshold for quantity or duration of opioid supply at the two time points e.g. need to fill more than 30 days’ supply at either time points.
- Studies with a POU definition that specified the need for any opioid exposure in addition to the two-time frames mentioned above e.g. If a further prescription needs to be collected between 30 and 90 days after the event.
- Studies that did not specify or include a fixed period for the assessment of POU i.e. open follow-up period after 90 days.
- Studies that only included patients undergone dental, or aesthetics/body contouring procedures due to heterogeneity in pain management and patient population.

Exposure to opioids was defined as any evidence that the patient may have received opioids from either prescription or community pharmacy dispensing records, or from patient interviews during the follow-up period.

### Study review and classification

EndNote Library (EndNote X9 Thomson Reuters, New York, NY, US) and Rayyan (http://rayyan.qcri.org) [14] were used to manage the citations. Two researchers (JG, AHYC) screened the titles and abstracts of all identified citations; full texts were obtained for any potentially eligible studies or abstracts that did not have sufficient information for review. Studies that did not meet the inclusion criteria or had reasons for exclusion at this review stage were not reviewed further, with reasons for exclusion documented. Any study was classified as trauma-related if trauma was the main cause of hospitalisation (e.g. Ankle fracture) irrespective of the occurrence of any surgery. Otherwise, any study was considered to be surgical-related, if surgery was the main exposure.

### Data extraction

We developed a standardised pilot-tested data extraction form (Microsoft Excel 2023, Microsoft Corporation, Washington, US). The following variables from each of the studies were extracted:

- General study information (author, year of publication, country of origin)
- Study design (age restriction, inclusion/exclusion criteria)
- Type and years of data used (institutional, linked administrative data)
- Study population (surgery or trauma, related specialities, number of participants in primary analysis)
- POU definition
- Incidence of POU
- Risk factors for POU

### Quality assessment and risk of *bias*

The Newcastle–Ottawa Scale (NOS) for cohort studies was used to assess the risk of bias for studies. Good quality studies had three or four stars in the selection domain *and* one or two stars in the comparability domain *and* 2 or 3 stars in the outcome domain. Fair quality studies had two stars in the selection domain *and* 1 or 2 stars in the comparability domain *and* 2 or 3 stars in the outcome or the exposure domain. Poor quality studies had zero or one star in either the selection *or* the outcome domain *or* zero stars in the compatibility domain [15]. The NOS has been widely used to assess the risk of bias in other systematic reviews, and it has been shown to correlate well with the Cochrane Collaboration's recommended risk of bias assessment tool, the Robins-I [6, 16, 17].

### Analysis

Data were synthesised narratively. The main outcome on the incidence of POU was reported as the percentage and absolute crude number of the final cohort included for analysis. We examined potential risk factors based on several categories: sociodemographic, baseline comorbidities, baseline medication use, inpatient variables, and any prescribing practice on discharge. The effect size of risk factors for POU was reported as aORs with 95% CI if P < 0.05, where suitable. Due to the heterogeneity in study characteristics, patient groups and measurement of risk factors, a meta-analysis was not able to be performed.

## Results

**Fig. 1:**
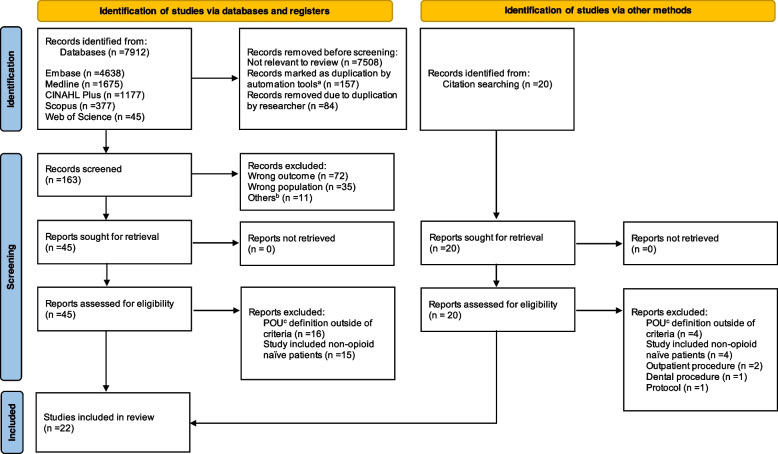
Flowchart for study selection. **a** Automated removal of duplicates done via Rayyan. **b** Entry was a review (*n* = 7), meta-analyses (*n* = 3), or consensus statement (*n* = 1). **c** Persistent opioid use

A total of 7,932 hits were retrieved from the initial search. Sixty-five entries were eligible for full-text screening. After full text review, another 43 references were excluded, leaving a final of 22 studies which met the inclusion criteria (Fig. 1).

The characteristics of included studies are presented in Table 1. The 22 studies included 20 surgical and two trauma studies. All studies were retrospective and most (21/22) studies used medico-administrative data (e.g. Medicare or commercial insurance claims). Out of 20 surgical studies, 18 originated from the US. Both trauma studies were conducted in the US.

**Table 1:** Characteristics of all included studies

Study (author, year and country of origin)	Study design (retrospective/prospective, main inclusion and exclusion criteria)	enrolment period and data type	POU definition	Study population (surgery/trauma, subspecialty)	Number of patients (in analysis, POU incidence)	Significant risk factors (adjusted odds ratios (aOR); 95% confidence interval (CI)
Johnson et al 2016 United States [18]	Retrospective Age: ≥ 18 Exclusions: Opioid exposure 11 months prior Diagnosis of opioid dependence or abuse 12 months prior	January 2010 to December 2012 Medico-administrative (MarketScan)	Opioid dispensed 30 days before and 14 days after surgery, additional dispensing of any opioids 90 to 180 days	Trauma Hand	59,725 POU: 6,272 (10.5%)	≥ 65 age (vs 18–34): aOR = 0.8; 95% CI 0.7–0.9 Female: aOR = 1.10; 95% CI 1.00–1.10 ≥ 70 ka income: aOR = 0.70; 95% CI 0.60–0.90 Elixhauser comorbidity index: 2 (vs 0 or 1): aOR = 1.30; 95% CI 1.30–1.50 > 3 (vs 0 or 1): aOR = 2.20; 95% CI 2.00–2.30 Mental health disorders: aOR = 1.10; 95% CI 1.00–1.20 Tobacco abuse: aOR = 1.60; 95% CI 1.00–2.60
Brummet et al. 2017 United States [19]	Retrospective Age: 18 to 64 Exclusions: Opioid exposure 11 months prior Sequential anesthsia postoperatively Length of stay > 30 days	January 2012 to June 2015 Medico-administrative (Clinformatic)	Opioid dispensed 30 days before and 14 days after discharge, additional dispensing of any opioids 90 to 180 days	Surgery 13 elective procedures	36,177 POU: 2,187 (6.0%)	40–49 age (vs 18–29): aOR = 0.72; 95% CI 0.61–0.84 High school Education (vs college): aOR = 1.22 95% CI 1.04–1.43 Charlson comorbidity: aOR = 1.10; 95% CI 1.08–1.13 History of tobacco use: aOR = 1.35; 95% CI 1.21–1.49 Anxiety: aOR = 1.25; 95% CI 1.10–1.42 Mood: aOR = 1.15; 95% CI 1.01–1.30 Alcohol/substance abuse: aOR = 1.34; 95% CI 1.05–1.72 Pain disorders: aOR = 1.39; 95% CI 1.26–1.54 Back pain: aOR = 1.57; 95% CI 1.42–1.75 Neck pain: aOR = 1.22; 95% CI 1.07–1.39 Arthritis: aOR = 1.93; 95% CI 1.71–2.19 Perioperative OME^b^ received ≥ 300 mg: aOR = 1.14; 95% CI 1.03–1.27
Lee et al 2017 United States [20]	Retrospective Age: ≥ 18 Exclusions: Opioid exposure 11 months prior Length of stay > 30 days Subsequent procedure within 6 months Discharged to home hospice care Died during hospitalisation	January 2010 to June 2014 Medico-administrative (MarketScan)	Opioid dispensed 30 days before and 14 days after discharge, additional dispensing of any opioids 90 to 180 days	Surgery Cancer-related surgery	39,877 POU: 4,159 (10.4%)	Breast cancer surgery: Age: aOR = 0.99; 95% CI 0.99–1.00 SUD^c^: aOR = 1.39; 95% CI 1.13–1.70 Elixhauser comorbidity score: aOR = 1.02; 95% CI 1.01–1.02 Median income (vs 60-70 k) ≥ 70 k: aOR = 0.87; 95% CI 0.79–0.97 Colorectal surgery: Elixhauser comorbidity score: aOR = 1.02; 1.01- 1.03 Median income (vs 60-70 k) ≥ 70 k: aOR = 0.75; 95% CI 0.61–0.93 Thoracic surgery: Female: aOR = 0.73; 95% CI 0.59–0.91
Harbaugh et al 2018 United States [21]	Retrospective Age: 13 to 21 Exclusions Opioid exposure 11 months prior Subsequent procedure within 6 months	January 2010 to December 2014 Medico-administrative (MarketScan)	Opioid dispensed 30 days before and 14 days after surgery, additional dispensing of any opioids 90 to 180 days	Surgery 13 Paediatric specialties	88,637 POU: 4,267 (4.8%)	Age: aOR = 1.07; 95% CI 1.05–1.08 Female: aOR = 1.22; 95% CI 1.14–1.31 SUD: aOR = 1.41; 95% CI 1.12–1.77 Chronic pain: aOR = 1.48; 95% CI 1.33–1.66
Bennet et al 2018 United States [22]	Retrospective Age: 8 to 25 Exclusions Opioid exposure 11 months prior to surgery Subsequent procedure within 6 months	January 2010 to December 2014 Medico-administrative (MarketScan)	Opioid dispensed 30 days before and 14 days after surgery, additional dispensing of any opioids 90 to 180 days	Surgery Cleft-related	2,034 POU: 90 (4.4%)	Age: aOR = 1.11; 95% CI 1.04–1.17 Paediatric chronic conditions Gastrointestinal: aOR = 7.37; 95% CI 1.49–36.54
Brescia et al 2019 United States [23]	Retrospective Age: ≥ 18 Exclusions Opioid exposure 11 months prior to surgery Length of stay > 30 days Subsequent procedure within 6 months Discharged to home hospice care Died during hospitalisation	January 2010 to June 2014 Medico-administrative (MarketScan)	Opioid dispensed 30 days before and 14 days after discharge, additional dispensing of any opioids 90 to 180 days	Surgery Lung-resection	3,026 POU: 424 (14.0%)	Age < 64: aOR = 1.30; 95% CI 1.05–1.62 Male: aOR = 1.39; 95% CI 1.12–1.72 Length of stay > 5 days: aOR = 1.30; 95% CI 1.04–1.63
Brescia et al 2019 United States [24]	Retrospective Age: ≥ 65 Exclusions Opioid exposure 11 months prior to surgery Length of stay > 30 days Subsequent procedure within 6 months	January 2009 to June 2015 Medico-administrative (Medicare)	Opioid dispensed 30 days before and 14 days after discharge, additional dispensing of any opioids 90 to 180 days	Surgery Cardiothoracic	24,549 POU: 3,153 (12.8%)	Length of stay: aOR = 1.03; 95% CI 1.02–1.04 Perioperative OME: aOR = 1.00; 95% CI 1.00–1.00 Age: aOR = 0.99; 95% CI 0.98–1.00 Female: aOR = 1.14; 95% CI 1.04–1.25 African American: aOR = 1.50; 95% CI 1.04–1.25 Charlson comorbidity (vs 0,1,2) 3,4: aOR = 1.14; 95% CI 1.02–1.26 5,6: aOR = 1.31; 95 CI 1.16–1.48 Tobacco use: aOR = 1.10; 95% CI 1.01–1.20 substance use: aOR = 1.31; 95% CI 1.05–1.63 Back pain: aOR = 1.27; 95% CI 1.16–1.38 Arthritis: aOR = 1.13; 95% CI 1.04–1.23 Other pain disorder: aOR = 1.10; 95% CI 1.02–1.19
Thiels et al 2019 United States [25]	Retrospective Age: ≥ 65 Exclusions Opioid exposure 6 months prior Treatment for opioid use disorder by buprenorphine or methadone 90 days after surgery Multiple procedure on the same day Length of stay > 7 days Admitted as inpatient 1 day before surgery Cancer patients receiving non-cancer surgeries Patients receiving hospice care Discharged to a skilled nursing facility on the day of discharge	January 2009 to June 2018 Medico-administrative (Optumlab)	Opioid dispensed 7 days before and after discharge, additional dispensing of any opioids 90 to 180 days	Surgery 20 commonly performed procedures	444,764 POU: 31,431 (7.1%)	Female: aOR = 1.22; 95% CI 1.19–1.26 Ethnicity (vs white): African American: aOR = 1.25; 95% CI 1.20–1.30, Asian: aOR = 0.80; 95% CI 0.72–0.88 Age 45–54 (vs 18–24): aOR = 1.28; 95% CI 1.18–1.38) Age 65–74: aOR = 1.29; 95% CI 1.17–1.43 OME 500 (vs 1–199): aOR = 1.20; 95% CI 1.14–1.26 Alcohol misuse: aOR = 1.45; 95% CI 1.23–1.78 Substance misuse aOR = 1.47; 95% CI 1.11–1.95 Depression: aOR = 1.41; 95% CI 1.32–1.50
Roughead et al 2019 Australia [26]	Retrospective Age: 18 to 100 Exclusions Opioid exposure 6 months prior Died during hospital admission	January 2014 to December 2015 Medico-administrative (Veteran’s Affairs)	Opioid dispensed 2 days before and 7 days after discharge, additional dispensing of any opioids 90 to 365 days	Surgery 14 different specialities	3,907 POU: 153 (3.9%)	N/A
Berger et al 2019 United States [27]	Retrospective Age: Adult Exclusions: Opioid exposure 150 days prior Subsequent procedure within 30 days or 365 days of index procedure Greater than 2 opioid refills prior Discharged to a facility	October 2010 to September 2014 Medico-administrative (Optumlab)	Opioid dispensed 30 days before and 7 days after discharge, additional dispensing of any opioids 91 to 180 days	Surgery Urological	38,689 POU: 2,399 (6.2%)	Female: aOR = 1.32; 95% CI 1.13–1.55 Length of stay: aOR = 1.05; 95% CI 1.02–1.08 Elixhauser comorbidity ≥ 3: aOR = 1.42; 95% CI 1.22–1.66 Mental health disorders: aOR = 1.37; 95% CI 1.23–1.52 Chronic Pain: aOR = 1.40; 95% CI 1.11–1.78
Gil et al 2019 United States [28]	Retrospective Age: ≥ 18 Exclusions Opioid exposure 11 months prior Subequent procedure within 6 months Length of stay > 30 days Not discharged home	January 2010 to March 2015 Medico-administrative (MarketScan)	Opioid dispensed 30 days before and 14 days after surgery, additional dispensing of any opioids 90 to 180 days	Surgery Orthopaedic	104,154 POU: 8,686 (8.3%)	Age 18–29 (vs 50–59): aOR = 0.68; 95% CI 0.61–0.76 Age > 70: aOR = 0.90; 95% CI 0.81–0.99 Female: aOR = 1.26; 95% CI 1.20–1.32 Median income 50 k-59 k (vs > 70 K): aOR = 1.10; 95% CI 1.03–1.17 Anxiety: aOR = 1.17; 95% CI 1.07–1.28 Charlson comorbidity: aOR = 1.11; 95% CI 1.09–1.13 Mood disorder: aOR = 1.29; 95% CI 1.19–1.40 Suicide/self-harm: aOR = 1.97; 1.29–3.42 Alcohol misuse: aOR = 1.57; 95% CI 1.30–1.89 Back pain: aOR = 1.09; 95% CI 1.03–1.15 Neck Pain: aOR = 1.12; 95% CI 1.05–1.19 Perioperative OME (> 743 mg): aOR = 2.00; 95% CI 1.91–2.10
Wright et al. 2019 United States [29]	Retrospective Age: 18 to 64 Exclusions Opioid exposure 11 months prior Subequent procedure within 6 months	2009 to 2016 Medico-administrative (MarketScan)	Opioid dispensed 30 days before and 14 days after surgery, additional dispensing of any opioids 90 to 180 days	Surgery Gynaecology	438,039 POU: 29,643 (6.8%)	Age 18–29 (vs 40–49): aOR = 1.22; 95% CI 1.17–1.28 Elixhauser comorbiditiy > 2: aOR = 1.20; 95% CI1.14–1.25) Mental health disorders: aOR = 1.14; 95% CI 1.09–1.19 Anxiety: aOR = 1.15; 95% CI 1.11–1.20 SUD: aOR = 1.41; 95% CI 1.34–1.48 Total perioperative OME 200–299 (vs 135): aOR = 1.16; 95% CI 1.10–1.22
Bicket et al 2019 United States [30]	Retrospective Age: 18 to 64 Exclusions Opioid exposure 11 months prior Additional procedure 30 days before to 14 days after	January 2010 to December 2015 Medico-administrative (MarketScan)	Opioid dispensed 30 days before and 14 days after surgery, additional dispensing of any opioids 90 to 180 days	Surgery Unspecified	912,882 POU: 93,159 (10.2%)	Age 40–49 (vs 18–29): aOR = 1.18; 95% CI 1.15–1.21 Female: aOR = 1.15; 95% CI 1.13–1.17 Smoking: aOR = 1.25; 95% CI 1.22–1.29 Charlson: aOR = 1.16; 95% CI 1.15–1.17 Anxiety: aOR = 1.15; 95% CI 1.13–1.18 Mood: aOR = 1.27; 95% CI 1.24–1.30 Alcohol misuse: aOR = 1.22; 95% CI 1.13–1.30 substance misuse: aOR = 1.40; 95% CI 1.30–1.52 Back pain: aOR = 1.18; 95% CI 1.16–1.20 Neck pain: aOR = 1.07; 95% CI 1.05–1.10) Arthritis: aOR = 1.20; 95% CI 1.18–1.22) Total perioperative OME: aOR = 1.05; 95% CI 1.03–1.07
Gossett et al 2019 United States [31]	Retrospective Age: 18 to 64 Exclusions Opioid exposure 12 months prior Multiple procedure Subsequent procedure within 6 months Admitted with multiple trauma	January 2009 to June 2016 Medico-administrative (Optumlab)	Opioid dispensed 14 days before and seven days after surgery, additional dispensing of any opioids 91 to 180 days	Trauma Ankle fracture	13,088 POU: 1,061 (8.1%)	Female: aOR = 1.16; 95% CI 1.01–1.34 Income > 100 K (vs 60 K-74 K): aOR = 0.73; 95% CI 0.58–0.90 Total perioperative OME (> 650 mg): aOR = 1.56; 95% CI 1.34–1.82 Charlson score > 5 (vs 0): aOR = 1.92; 95% CI 1.19–3.09 Tobacco use: aOR = 1.37; 95% CI 1.17–1.60 Anxiety: aOR = 1.57; 95% CI 1.30–1.90 Mood: aOR = 1.25; 95% CI 1.05–1.50 Substance misuse: aOR = 1.33; 95% CI 1.01–1.75 Arthritis: aOR = 0.56; 95% CI 0.43–0.72
Ward et al 2020 United States [32]	Retrospective Age: ≤ 18 Exclusions Opioid exposure 90 days prior Without anesthesia code Additional anesthetic code within 365 days Length of stay ≥ 30 days	December 2002 to December 2017 Medico-administrative (Optumlab)	Opioid dispensed 30 days before and 14 days after surgery, additional dispensing of any opioids 90 to 180 days	Surgery Paediatric	173,388 POU: 3,523 (2.0%)	Age 2–6: Ethnicity Asian (vs European): aOR = 0.24; 95% CI 0.06–0.97, African American: aOR = 1.50; 95% CI 1.00–2.20 Age 6–12: Asian: aOR = 0.33; 95% CI 0.12–0.90 Age 12–18: Male: aOR = 0.82; 95% CI 0.75–0.89 Asian: aOR = 0.51; 95% CI 0.34–0.77 Mood disorder: aOR = 1.4; 95% CI 1.2–1.6 SUD: aOR = 1.30; 95% CI 1.00–1.70 Chronic Pain: aOR = 1.40; 95% CI 1.20–1.60
Brown et al 2020 United States [33]	Retrospective Age: ≥ 18 Exclusions Opioid exposure 6 months prior Palliation within 6 months after surgery Subsequent anesthesia 6 months after surgery Preoperative metastatic disease	January 2004 to December 2016 Medico-administrative (Optumlab)	Opioid dispensed 14 days after surgery, additional dispensing of any opioids 90 to 180 days	Surgery Cardiac	35,817 POU: 3,430 (9.6%)	Men: aOR = 0.87; 95% CI 0.79–0.97 Income 100 K (ref < 40 k): aOR = 0.87; 95% CI 0.77–0.99 Rheumatoid arthritis: aOR = 1.57; 95% CI 1.25–1.96 Chronic pain: aOR = 2.73; 95% CI 2.10–3.56 Alcohol misuse: aOR = 1.56; 95% CI 1.23–2.00 Muscle relaxant use: aOR = 1.74; 95% CI 1.51–2.02 Benzodiazepine use: aOR = 1.71; 95% CI 1.52–1.91 Length of stay: aOR = 1.03; 95% CI 1.01–1.04
Clement et al 2020 United States [34]	Retrospective Age: Adult Exclusions Opioid exposure 365 to 31 days prior Died in hospital Discharged to hospice Underwent concomitant coronary artery bypass grafting Subsequent surgery within 180 days	2014 to 2016 Medico-administrative (MarketScan)	Opioid dispensed 30 days before and 14 days after surgery, additional dispensing of any opioids 90 to 180 days	Surgery Heart valve	3,404 POU: 188 (5.5%)	Perioperative OME increase: aOR = 1.01; 95% CI 1.01–1.01 1% increase for every 10 mg OME prescribed
Clement et al 2020 United States [35]	Retrospective Age: Adult Exclusions Opioid exposure 365 to 31 days prior Died in hospital Discharged to hospice Subsequent major surgery within 180 days Descending and thoracoabdominal aortic repairs	January 2012 to December 2017 Medico-administrative (MarketScan)	Opioid dispensed 30 days before and 14 days after surgery, additional dispensing of any opioids 90 to 180 days	Surgery Open thoracic aortic	3,204 POU: 169 (5.3%)	Perioperative OME increase: aOR = 1.01; 95% CI 1.00–1.01 1% increase for every 10 mg OME prescribed nicotine use: aOR = 2.09; 95% CI 1.35–3.25
Clement et al 2020 United States [36]	Retrospective Age: Adult Exclusions Opioid exposure 365 to 31 days prior Died in hospital Discharged to hospice Subsequent surgery within 180 days Length of stay > 30 days	2014 to 2016 Medico-administrative (MarketScan)	Opioid dispensed 30 days before and 14 days after surgery, additional dispensing of any opioids 90 to 180 days	Surgery Coronary bypass	7,292 POU: 590 (8.1%)	Female: aOR = 1.30; 95% CI 1.05–1.61 Anxiety: aOR = 1.40; 95% CI 1.09–1.81 tobacco use: aOR = 1.34; 95% CI 1.08–1.65 Substance misuse: aOR = 1.99; 95% CI 1.08–1.65 Length of stay: aOR = 1.02; 95% CI 0.99–1.05 Perioperative OME increase: aOR = 1.02; 95% CI 1.01–1.02 2% increase for every 10 mg OME prescribed
Santosa et al 2020 United States [37]	Retrospective Age: ≥ 65 Exclusions Opioid exposure 11 months prior Additional procedure 180 days after	January 2009 to June 2015 Medico-administrative (Medicare)	Opioid dispensed 30 days before and 14 days after surgery, additional dispensing of any opioids 90 to 180 days	Surgery 13 selected procedures	81,839 POU: 8,021 (9.8%)	Charlson comorbidity ≥ 5 (vs 0): aOR = 1.71; 95% CI 1.58–1.84 Mood disorders: aOR = 1.16; 95% CI 1.09–1.24) Suicide: aOR = 1.60; 95% CI 1.05–2.44 Alcohol misuse: aOR = 1.38; 95% CI 1.20–1.59 Back Pain: aOR = 1.16; 95% CI 1.07–1.27 Total Perioperative OME (> 300 mg): aOR = 1.44; 95% CI 1.37–1.52 Hypnotic use: aOR = 1.24; 95% CI 1.14–1.35 Benzodiazepine use: aOR = 1.24; 95% CI 1.14–1.35 African American (vs Caucasian) aOR = 1.23; 95% CI 1.12–1.36 Long-acting opioids on discharge: aOR = 2.87; 95% CI 2.18, 3.76 Co-prescribed benzodiazepine on discharge: aOR = 4.83; 95% CI 4.08–5.71
Delaney et al 2020 United States [38]	Retrospective Age: ≥ 65 Exclusions Opioid exposure 11 months prior Discharged to facility (other than home) Length of stay > 30 days Additional anaesthetic code 180 days after	January 2013 to June 2016 Medico-administrative (Medicare)	Opioid dispensed 30 days before admission and 3 days after discharge, additional dispensing of any opioids 91 to 180 days	Surgery Orthopaedic	1,403 POU: 97 (6.9%)	Total OME on discharge 3rd quartile, 497 OME (vs 1st quartile): aOR = 2.91; 95% CI 1.53–5.51
Beyene et al 2022 New Zealand [39]	Retrospective Age: ≥ 18 Exclusions Opioid exposure 90 days prior Subsequent surgery or trauma 180 days after Discharged to facility	January to December 2019 Institutional data	Opioid dispensed 7 after discharge, additional dispensing of any opioids 91 to 180 days	Surgery General surgery	401 POU: 38 (9.5%)	Female: aOR = 2.29; 95% CI 1.07–4.90 Length of stay > 24 h: aOR = 3.07; 95% CI 1.30–7.21 Perioperative OME > 100: aOR = 3.04; 95% CI 1.25–7.40 Surgical severity: Category 3 severity (vs 1): aOR = 0.17; 95% CI 0.01–0.53

^a^*K* 1,000 in US dollar currency e.g. $US70,000

^b^*OME* Oral morphine equivalent

^c^*SUD* Substance use disorder

### Incidence of POU

In the 19 surgical studies with patients 18 years and over, the reported incidence of POU among opioid naïve users varied between 3.9% (*n* = 153) to 14.0% (*n* = 424) [19–23, 27, 28, 30, 33–36, 39]. However, among the 19 studies, two included predominantly younger patients with lower incidences of POU –*Harbaugh *et al. included patients aged between 13 to 21 [21], whilst *Bennet *et al. included patients aged between 8 to 25 [22]. The incidence of POU was similar in these two studies at 4.8% (*n* = 4,267) and 4.4% (*n* = 90) respectively [21, 22]. In the single surgical study with patients under 18, the incidence of POU was found to be 2.0% (*n* = 3,523) [32]. In surgical studies that included a range of surgical specialties and in patients 18 years and over, the incidence of POU was 3.9% (*n* = 153) to 10.2% (*n* = 93,159) [19, 25, 26, 30, 37]. In cardiac surgery amongst patient 18 years and over, the incidence of POU was 5.2% (*n* = 169) to 12.8% (*n* = 3,153) [23, 33–36]. The surgical specialty (and incidence of POU) in other studies with patients 18 years and over were in cancer-related surgery with curative intent (10.0% [*n* = 4,159] to 14.0% [*n* = 424]) [20, 23], urological (6.2% [*n* = 2,399]) [27], orthopaedic (6.9% [*n* = 97] to 8.3% [*n* = 8,686]) [28, 38], gynaecology (6.8% [*n* = 29,643]) [29], and general surgery (9.5% [*n* = 38]) [39]. In the two trauma studies, the incidence of POU was between 8.1% (*n* = 1,061) and 10.5% (*n* = 6,272) [18, 31].

### Sociodemographic risk factors of POU

Age as a potential risk factor of POU was included in the final analysis for 19 of the 20 surgical studies. However, age was not consistently identified as a significant risk factor. Of these, 63.2% (12/19) surgical studies found age to be a significant risk factor for POU. Over half (7/12) of the studies found that patients of older age had high risk of POU [21–25, 30, 33]. Comparison of effect size between studies was difficult given each study had arbitrarily defined their age groups and used different age groups as the reference. One exception was in a gynaecology study where younger patients aged 18 to 29 years were reported to have the highest risk of becoming persistent opioid users compared to other age groups (aOR = 1.22; 95% CI 1.17–1.29, *P* < 0.001) [29]. Another study conducted in orthopaedic patients found mixed results with respect to age, when compared to age 50–59, age 18–29 (aOR = 0.68; 95% CI 0.61–0.76, *P* < 0.001) and over 70 (aOR = 0.90; 95% CI 0.81–0.99, *P* = 0.035) both had decreased odds of POU [28]. In the trauma studies, all studies included age as a risk factor but only one study found age to have a statistically significant association with POU [18]. The study found those aged over 65 years compared to 18 to 34 years had lower odds of becoming a persistent opioid user (aOR = 0.80; 95% CI 0.70–0.90, *P* = 0.001) [18].

A total of 90.0% (18/20) of surgical studies included sex as a risk factor for POU, half (9/18) of these studies found sex was a significant risk factor. Of these, 88.9% (8/9) of the studies found that females were at higher odds of developing POU with aORs between 1.14 and 2.29 [21, 24, 27, 28, 30, 35, 36, 39]. Both trauma studies found females to have a small increase in odds of developing POU with aOR of 1.10 and 1.16 respectively [18, 31].

A total of 35% (7/20) of the surgical studies included ethnicity as a risk factor for POU, and 42.9% (3/7) of those studies found significant differences in developing POU between ethnicities. When compared to Europeans, African Americans were at higher odds of becoming POU in all three studies aOR between 1.23 and 1.50 [24, 32, 37]. Only one trauma study included ethnicity as a risk factor but it was not found to be statistically significant [31]. Over one third (7/20) of surgical studies that included annual income as a risk factor of POU, 42.9% (3/7) surgical studies found that income was a statistically significant variable, and annual income higher than $USD 70,000 was negatively associated with POU with aOR between 0.75 and 0.90 [20, 28, 33]. Similarly, both trauma studies found annual income greater than $USD 70,000 was associated with significantly reduced risk of POU aOR of 0.70 and 0.73 respectively [18, 31].

### Baseline comorbidities

Over a third (7/20) of surgical studies that included a comorbidity index such as Charlson or Elixhauser, as a measure of a patient’s overall comorbidity burden, and as a risk factor of POU; 85.7% (6/7) studies reported that a greater comorbidity burden was positively associated with POU, whilst one did not find that overall comorbidity burden was a significant risk factor [19, 24, 28, 34, 36, 37]. Out of the studies that found a greater comorbidity burden was positively associated with POU, 83.3% (5/6) of studies used the Charlson index as a measure of overall comorbidity burden; however different approaches on how comorbidity was measured differed across studies. In the one surgical study using the Elixhauser comorbidity index, an increased score (indicating increased comorbidity burden) was associated with a 2.0% increase in the risk of POU per additional point on the index (*P* < 0.001) [20]. Similarly, both trauma studies reported that greater comorbidity burden was also associated with increased odds of POU. However, the two studies were not comparable as they used different indices to measure overall comorbidity burden; *Johnson *et al*.* used the Elixhauser index and *Gossett *et al*.* used the Charlson index [18, 31].

Baseline diagnosis of mental health and pain-related disorders were the most common comorbidities included in the analysis for POU in both surgical and trauma patients. Of the 20 surgical studies included, 70.0% (14/20) of the studies had at least one mental health disorder [19–21, 23–25, 27–30, 32, 36–38] and half (10/20) of the surgical studies included at least one pain-related disorder [19, 21, 24, 27, 28, 30, 32, 33, 37, 38]. The most commonly reported mental health disorder was previous substance use disorder (SUD) from 57.1% (8/14) of the surgical studies, which all had positive associations with POU; aOR was between 1.30 and 1.99 [19–21, 24, 25, 30, 32, 36]. Less than half (4/10) of surgical studies, that included any pain-related disorder found back pain was the most consistently positive significant predictor for POU; aOR between 1.09 and 1.27 [24, 28, 30, 37].

The two trauma studies included both mental health and pain-related disorders [18, 31]. SUD was also the most commonly reported risk factor related to mental health disorder among trauma studies, but only one study found it to be a significant risk factor of POU (aOR = 1.33; 95% CI 1.01–1.75, *P* = 0.040) [31]. Both trauma studies also included baseline pain disorders, *Johnson *et al*.* found it was a significant risk factor [18] and *Gossett *et al*.* found it was negatively associated with POU (aOR = 0.56; 95% CI 0.43–0.72, *P* < 0.001) [31]. Only arthritis was included under the category of pain disorders in the study by *Gossett *et al. [31].

### Baseline medication use

Medication use prior to hospital admission did not commonly feature as an independent risk factor of POU in either surgery or trauma studies examined. Only 10.0% (2/20) of surgical studies examined baseline medication use, and the studies found hypnotics such as benzodiazepines increased the odds of POU in both studies with aOR of 1.24 and 1.71 [33, 37]. No trauma studies included any baseline medication use as a risk factor of POU.

### Inpatient variables

Operation type was commonly included as a risk factor for POU in surgical studies, but given the heterogeneity in how different operations were defined between studies, it was not possible to group these for comparison. Hospital length of stay (LOS) was included as a risk factor in over a third (7/20) of surgical studies [23, 24, 27, 33, 34, 36, 39], and 71.4% (5/7) studies found increasing LOS to be a positive risk factor of POU [23, 24, 27, 33, 36]. Four studies defined this variable similarly as increased in odds per each additional day of LOS and this was reflected in their closely aligned effect size (aOR between 1.02 to 1.05) [24, 27, 33, 36]. The study by *Brescia *et al*.* defines LOS as a dichotomous variable of more than five days (vs less than five days) and found aOR = 1.30; 95% CI 1.04–1.63, *P* < 0.001 [23]. LOS was not included as a risk factor for any trauma studies.

### Opioid prescribing practices after the surgical or trauma event

Of the surgical studies included 65.0% (13/20) had included any opioid prescribing practices after surgery as a risk factor for POU. This was analysed by considering the total amount of opioid dose on the discharge prescription, converting this to an oral morphine equivalent (OME)- in milligrams, and consider the supply during the perioperative period. This was a risk factor that was included in all 13 studies [19, 20, 24, 25, 28–30, 34–39]. All studies found that increasing dosages of opioids prescribed during the perioperative period were associated with increased odds of POU. However, there were large variations between studies on how the POU variable was defined and analysed, making comparisons between studies difficult. One trauma study by *Gossett *et al*.* found that patients who were prescribed larger quantities of opioids (more than 650 mg in OME) after discharge were associated with increased odds of becoming a persistent opioid user (aOR = 1.56; 95% CI 1.34–1.82) [31].

### Risk of *bias* assessment

The overall risk of bias across all studies were low (21 out of 22 studies), with only one surgical study being classified being at high risk of bias [26]. In terms of selection bias, all studies scored between three to four stars (maximum being four stars). In the comparability category, only one surgical study scored the maximum two stars as it required the inclusion of marital status, and another surgical study scored zero stars as no confounders were included for the analysis of risk factors related to POU [26]. Lastly, in the outcome category, 19 out of the 22 studies received the maximum allocated three stars and three out of 22 receiving two stars. See Table 2 for scoring per item for included studies.

**Table 2:** Newcastle–Ottawa scale ratings for included studies

Study	Selection				Comparability	Outcome			Total score and quality
	Representativeness of exposed cohort (one star)	Selection of non-exposed cohort (one star)	Ascertainment of exposure (one start)	Outcome of interest does not present at start of star (one star)	One or two stars	Assessment of outcome (one star)	Length of follow up adequate for outcome (one star)	Adequacy of follow up (one star	
Bennet et al. [22]	*	*	*	*	*	*	*	*	8 Good
Berger et al. [27]	*	*	*	*	*	*	*	*	8 Good
Beyene et al. [39]	*	*	*	*	*	*	*		8 Good
Bicket et al. [30]	*	*	*	*	**	*	*		8 Good
Brescia et al. [23]	*	*	*	*	*	*	*	*	8 Good
Brescia et al. [24]	*	*	*	*	*	*	*	*	8 Good
Brown et al. [33]	*	*	*	*	*	*	*	*	8 Good
Brummet et al. [19]	*	*	*	*	*	*	*	*	8 Good
Clement et al. [36]	*	*	*	*	*	*	*	*	8 Good
Clement et al. [35]	*	*	*	*	*	*	*	*	8 Good
Clement et al. [34]	*	*	*	*	*	*	*	*	8 Good
Delaney et al. [38]	*	*	*	*	*	*	*	*	8 Good
Gil et al. [28]	*	*	*	*	*	*	*	*	8 Good
Gossett et al. [31]	*	*	*	*	*	*	*	*	8 Good
Harbaugh et al. [21]	*	*	*	*	*	*	*	*	8 Good
Johnson et al. [18]	*	*	*	*	*	*	*	*	8 Good
Lee et al. [20]	*	*	*	*	*	*	*	*	8 Good
Roughead et al. [26]	*	*	*	*		*	*		6 Poor
Santosa et al. [37]	*	*	*	*	*	*	*	*	8 Good
Thiels et al. [25]	*	*	*	*	*	*	*	*	8 Good
Ward et al. [32]	*	*	*	*	*	*	*	*	8 Good
Wright et al. [29]	*	*	*	*	*	*	*	*	8 Good

## Discussion

This review was a systematic review of the current literature on the topic of POU that has synthesised evidence in patients after surgery or trauma, without restriction to specific surgical specialties and inclusion of only opioid-naïve patients. The findings of this review align closely with other systematic reviews conducted in the surgical and trauma space [6, 7]. However, the review is the first to systematically synthesise and compare findings between opioid-naïve surgical and trauma patients to highlight differences in the incidence of POU and risk factors in the current literature. Being able to systematically review these risk factors provides an important foundation to inform future research to reduce POU. A total of 22 studies were included, comprising of 20 surgical studies and two trauma studies. The quality of the studies was generally of good quality; however, most studies were of U.S origin and used medico-administrative data. The review also highlights the need for further research related to opioid use and opioid-related adverse events in countries outside of North America. Overall, there were large differences in the incidence of POU observed across studies regardless of them being either surgical or trauma cohorts. In the surgical cohort, we observed that studies with an adult population had relatively higher incidences of POU than those with mostly paediatric and young adult populations. This may reflect prescribing practice, where opioids are used more conservatively in younger patients, as younger patients may have more potential to develop addiction behaviours [40], and dosing may be more difficult [41].

Among the reported sociodemographic variables, no variables had consistent association with POU in surgical or trauma cohorts. However, in baseline comorbidities, there was a close alignment of findings. Among the seven surgical studies that included a comorbidity index, 85.7% (6/7) studies found that increasing comorbidity burden increased the odds of POU. Similar findings were also reported in both trauma studies. This was unsurprising, given that higher comorbidity burdens have also been shown to correlate with worse surgical and trauma outcomes [42–45]. These outcomes may result in hospital readmission and surgical complications, increasing the likelihood of prolonged opioid exposure. SUD was associated with increased POU in over half of the surgical studies that included a mental health disorder. Comparatively, in trauma studies, half of the studies that included mental health disorder as a risk factor found SUD was a significant risk factor [18]. There are complex dynamics in pain management and SUD, and it has been shown patients with SUD are less likely to receive effective pain management in both emergency and postoperative settings as there are concerns for misuse [46]. Furthermore, the co-existence of addiction and pain may act synergistically to re-enforce symptoms of either component, resulting in a vicious cycle of using other substances to manage pain and vice versa, i.e. opioids for addiction [47]. Both complications will likely result in poor pain control and increased risks of POU.

Benzodiazepines and increased risk of POU were unique to surgical studies as no trauma studies included this variable as a risk factor, which represents a gap for future research. The indications for benzodiazepines are broad, including anticonvulsant, anxiolytic, sedative, and muscle relaxant. Patients taking concomitant benzodiazepine at the time of surgery may indicate a history of anxiety, which has also been shown to increase odds of POU [29, 30]. Patients with anxiety may have higher chances of catastrophising pain, which may result in increased opioid need and chronic pain post-surgery [48]. It should also be noted that the co-prescribing of benzodiazepine and opioids is not recommended, as it may increase the risk of overdose via suppressing airways and impairing cognitive functions [49]. Of the surgical studies, 71.4% (5/7) of the studies found that longer LOS was associated with increased odds of POU, and this was consistent regardless of other indicators of operation severity or invasiveness (open vs laparoscopic) [24, 39]. As LOS is a proxy for hospital resource use, a higher LOS may indicate postoperative complications such as a need for additional surgery or admission to intensive care [50, 51]. Thus, greater LOS may result in greater analgesic need and increased complexity in pain management. Higher opioid amount during the perioperative and post-trauma period, as indicated via OME, was consistently found to be a significant risk factor of POU. The OME prescribed in this period may represent a risk for greater ongoing analgesia need but also reflect an over-supply of opioids on discharge, which we cannot determine from this review.

This review has several limitations. The initial search excluded conference proceedings due to limited information that can be captured in the proceedings. In our piloted searches the definition of POU and opioid-naïve status of patients were not detailed enough to be assessed for inclusion. Some studies may be missed due to the exclusion of conference proceedings, which may alter our reporting of the incidence of POU and effect size of risk factors. We considered the study to be involving opioid-naïve patients if the study mentioned that its analysis was restricted to opioid-naïve patients, but this may not be representative of true opioid-naivety. The studies mostly considered patients to be opioid naïve based on the absence of recent pharmacy claims for opioid dispensing up to 12 months prior to the event. However, patients may be included in the study if they had been dispensed opioids prior to the 12 months look-back period and may have had a diagnosis of SUD (including opioids). Only two studies in this review also excluded patients with a previous diagnosis of opioid-related SUD or having been on treatment for opioid dependence, in addition to the absence of recent opioid dispensing [18, 25]. Thus, the inclusion of patients with a previous diagnosis of opioid-related SUD in some studies may overestimate the prevalence of POU. The POU definition used in our review was specific so that the studies included could be comparable, but as the definition was narrow it may have excluded potentially relevant studies. Recent meta-analysis on POU definitions found the incidence of POU can vary up to two-fold depending on the definition used [3]. Thus, given the already significant heterogeneity in study design, broader POU definitions may give rise to further incongruity of findings across studies [3]. The nature of studies included also had limitations, as studies were mostly of US origin using medico-administrative data. This may not reflect the health system and healthcare utilisation patterns of other countries. The medico-administrative studies used either prescribing or pharmacy dispensing data as a proxy for the actual consumption of opioids, which may not represent the actual use of opioids. The studies included also did not provide any indications as to why patients continued to use opioids beyond 90 days as it may be clinically indicated in some patients with complex postoperative recovery. Finally, due to the significant heterogeneity in study design, cohort selection and reporting of risk factors, a meta-analysis could not be performed. Whilst we found that the quality of the studies was mostly good but there was some evidence of bias in all studies particularly relating to comparability. Outcomes related to POU were beyond the scope of this review but as there has been growing interest in evaluating outcomes related to POU as a long-term prognostic marker of poor outcomes after surgery and trauma, this is an area for further research. Recent studies have reported that POU may be associated with increased risk of mortality and morbidity [11, 52].

## Conclusion

This was the first review that we are aware of to examine the incidence of POU among opioid naïve patients after surgery or trauma. The review found that a significant proportion of opioid-naïve patients 18 and over following exposure to opioids after hospitalisation for surgery or trauma, may develop POU. Our findings suggest that there may be risk factors that were consistently and independently associated with increased odds of POU, irrespective of surgery or trauma. These risk factors included having a history of mental health disorders, chronic pain disorders, higher comorbidity burden, baseline hypnotic use, increased LOS, or being prescribed higher doses of opioids during the perioperative and postoperative and post-trauma period. These risk factors may be considered by prescribers to assess the risk of POU after surgery or trauma, when continuing opioids after hospitalisation. The overall quality of studies included was good, and the review also found several gaps in the literature related to trauma patients, which represents opportunities for future research to explore all relevant patient and clinical variables as risk factors for POU.

### Supplementary Information

Supplementary Material 1.

## Data Availability

The datasets used and/or analysed during the current study are available from the corresponding author on reasonable request.
