# Supplementary material for: Incidence and risk factors of new persistent opioid use after surgery and trauma: A systematic review
Source: BMC Surg. 2024 Jul 16;24:210. doi: 10.1186/s12893-024-02494-0 (PMC11251237; doi:10.1186/s12893-024-02494-0)
Supplement: Supplementary file 1 — Supplementary Material 1. [file 12893_2024_2494_MOESM1_ESM.docx]

# Supplementary material

**Part 1: search terms and strategies**

- OVID Medline and Embase database: (EXP Analgesics, Opioid OR EXP morphine OR EXP oxycodone OR EXP methadone OR EXP fentanyl OR EXP codeine OR EXP tramadol or EXP pethidine OR morphine.tw OR oxycodone.tw OR fentanyl.tw OR codeine.tw or tramadol.tw OR pethidine.tw)) AND (EXP surgical procedures, operative OR EXP injuries OR surger$.tw OR (surgical adj2 procedure$).tw OR (musculoskeletal adj2 injur$).tw OR Trauma.tw OR (limb adj2 injur$).tw OR (Acute adj2 injur$).tw) AND (EXP opioid-related disorders OR (persistent adj2 opioid).tw OR (Chronic adj2 opioid).tw OR (opioid adj2 misuse).tw OR (opioid adj2 dependence).tw. A similar search was applied to OVID embase, altering the MESH term for ‘analgesics’ to ‘Analgesic agent’ but keeping all other terms the same.
- CINAL plus: (MH ‘Analgesics, opioid OR MH ‘codeine’ OR MH ‘Fentanyl’ OR MH ‘Methadone’ OR MH ‘morphine’ OR MH ‘Oxycodone’ OR MH ‘Tramadol’ OR MH ‘Meperidine’) OR Opioid*.tw OR Opiate*.tw OR Pain relief.tw OR Painkiller*.tw OR Pain Killer.tw Or analgesi*.tw Or morphine.tw OR oxycodone.tw OR fentanyl.tw OR Codeine.tw OR tramadol.tw OR pethidine.tw Or methadone.tw) AND (MH ‘Postoperative Pain’ OR MH ‘Postoperative care’ OR MH ‘Postoperative period’ OR MH ‘Wounds and injuries+’ OR Postoperative.tw OR after surgery OR surger*.tw OR Hospital discharge*.tw OR injur*.tw OR trauma*.tw) AND (MH ‘Opioid Epidemic’ OR MH Addiction OR Chronic Use.tw OR Persistent use.tw OR Opioid dependen*.tw)
- Web of science: (Opioid OR (Opioid and addiction) OR (Persistent and opioid) OR (Opioid and chronic)) AND (Surgery OR Injur$ OR Trauma) AND (outcome OR (Clinical AND outcome) OR (hospitalisation OR hospitalization))
- Scopus: (Opioid OR morphine OR oxycodone Or fentanyl OR methadone OR pethidine Or tramadol OR codeine OR dihydrocodeine) AND (Surge$ OR operation$ OR trauma OR injur$ OR (acute AND injur$) AND (Persistent OR chronic OR dependence)
